# Joubert syndrome presenting bilateral peroneal neuropathies: A case report

**DOI:** 10.1097/MD.0000000000037987

**Published:** 2024-04-26

**Authors:** Hyeong-Min Kim, Hyun-Seok Jo, Jae-Young Han, In-Sung Choi, Min-Keun Song, Hyeng-Kyu Park

**Affiliations:** a Department of Physical and Rehabilitation Medicine, Research Institute of Medical Sciences, Heart Research Center, Chonnam National University, Chonnam National University Medical School & Hospital, Gwangju City, Republic of Korea.

**Keywords:** case report, gait disturbance, Joubert syndrome, peripheral neuropathy, TMEM67, whole genome sequencing

## Abstract

**Rationale:**

Joubert syndrome (JS) is a rare genetic disorder that presents with various neurological symptoms, primarily involving central nervous system dysfunction. Considering the etiology of JS, peripheral nervous system abnormalities cannot be excluded; however, cases of JS accompanied by peripheral nervous system abnormalities have not yet been reported. Distinct radiological findings on brain magnetic resonance imaging were considered essential for the diagnosis of JS. However, recently, cases of JS with normal or nearly normal brain morphology have been reported. To date, there is no consensus on the most appropriate diagnostic method for JS when imaging-based diagnostic approach is challenging. This report describes the case of an adult patient who exhibited bilateral peroneal neuropathies and was finally diagnosed with JS through genetic testing.

**Patient concerns and diagnosis:**

A 27-year-old man visited our outpatient clinic due to a gait disturbance that started at a very young age. The patient exhibited difficulty maintaining balance, especially when walking slowly. Oculomotor apraxia was observed on ophthalmic evaluation. During diagnostic workups, including brain imaging and direct DNA sequencing, no conclusive findings were detected. Only nerve conduction studies revealed profound bilateral peroneal neuropathies. We performed whole genome sequencing to obtain a proper diagnosis and identify the gene mutation responsible for JS.

**Lessons:**

This case represents the first instance of peripheral nerve dysfunction in JS. Further research is needed to explore the association between JS and peripheral nervous system abnormalities. Detailed genetic testing may serve as a valuable tool for diagnosing JS when no prominent abnormalities are detected in brain imaging studies.

## 1. Introduction

Joubert syndrome (JS) is a rare genetic disorder, which is predominantly inherited in an autosomal recessive manner. Classic JS is characterized by clinical manifestations such as hypotonia, ataxia, oculomotor apraxia, and intellectual disability.^[1]^ In addition to classic neurological symptoms, JS exhibits broad phenotypic heterogeneity, including retinal, renal, hepatic, and oral–facial–digital symptoms, according to the subtype of JS.^[2–4]^ Various forms of JS have been reported to date, with novel phenotypes continuing to emerge in recent times.

JS is included in the group of disorders called ciliopathies. It is well known that the primary cilium is an important subcellular organelle in neural circuit development. Mutations affecting the gene encoding the primary cilium lead to various symptoms associated with central nervous system (CNS) dysfunction.^[5]^ The primary cilium has an important role in the CNS, but is also pivotal to the formation of the peripheral nervous system (PNS) in vivo.^[6]^ However, PNS abnormalities in patients with JS have yet to be reported.

The diagnosis of JS is traditionally based on typical clinical symptoms and characteristic radiological findings, including the “molar tooth sign.” However, recent research has revealed that some people with JS exhibit normal or nearly normal brain images.^[7]^ Genetic testing is widely used in the diagnosis of JS and has the potential to become a diagnostic gold standard in some atypical cases.^[8]^

Here we report an atypical JS case presenting with gait disturbance and bilateral peroneal neuropathies.

## 2. Case presentation

A 27-year-old man visited our outpatient clinic with a complaint of gait abnormality. He experienced involuntary external rotations of both legs during ambulation. Although he could not remember the exact onset of gait disturbance, he recognized that the symptoms were present in middle school.

The patient had visited the neurology department 7 years ago because of the gait abnormality, at which time, brain magnetic resonance imaging (MRI) was performed to identify the cause. The results revealed mild elongation of the superior cerebellar peduncles, but these findings were not considered pathological. Nerve conduction studies revealed bilateral peroneal neuropathies, but no trigger was identified as a likely cause of the condition. Direct DNA sequencing was performed to rule out early-onset isolated dystonia, and pathologic TOR1A gene mutation associated with early-onset isolated dystonia was not detected at that time. Without confirming the underlying pathology, the patient visited the orthopedic surgery department, where he underwent medial hamstring lengthening and derotational osteotomy to correct the torsional deformity of the tibia. The patient visited our department to investigate the underlying cause of the gait disturbance after corrective surgery.

**Figure 1.**
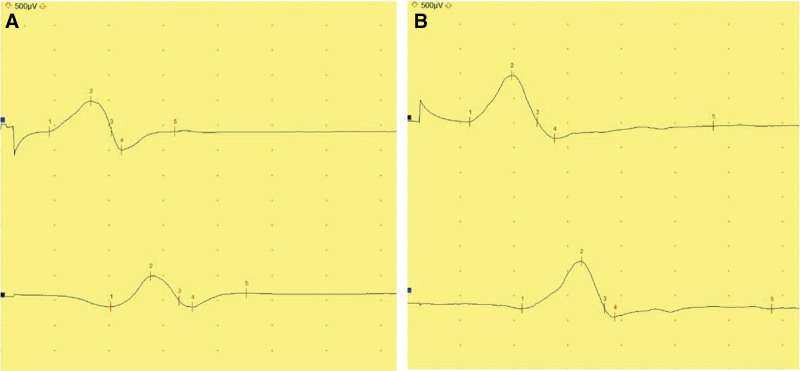
Peroneal motor nerve conduction study results. Compound muscle action potential of (A) right peroneal and (B) left peroneal nerves. Both responses are recorded over extensor digitorum brevis muscles.

**Figure 2.**
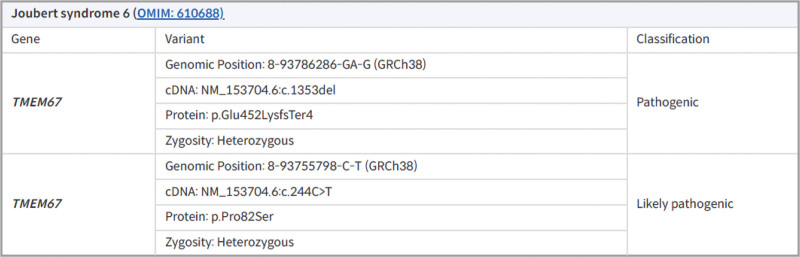
Whole genome sequencing report. In whole genome sequencing, heterozygous mutations in the TMEM67 gene associated with Joubert syndrome type 6 have been identified.

On neurological examination, the patient exhibited decreased muscular strength in all extremities (upper and lower limbs: MRC grade 4), and slight disequilibrium was suspected during the one-leg standing exercise (Berg Balance Scale: 50/56). Both legs were externally rotated during ambulation, and the patient dragged his left foot because of decreased ankle dorsiflexion. Although no prominent ataxia or upper motor neuron signs, such as increased spasticity or pathologic reflex, were observed, the patient felt an imbalance, particularly when walking slowly. Ophthalmic examination revealed decreased saccadic movement, convergence insufficiency, and lateral gaze limitation. Subsequently, follow-up nerve conduction studies were conducted. Motor and sensory nerve conduction studies of the median, ulnar, tibial, and sural nerves were normal. Recordings over the bilateral extensor digitorum brevis (EDB) muscles revealed low-amplitude peroneal responses, whereas those over the bilateral tibialis anterior muscles showed normal responses (Fig. 1, Table 1). Sensory nerve conduction studies of the superficial peroneal nerves showed normal responses. During needle electromyography, positive sharp waves and fibrillation potentials were observed in both EDB muscles. The right EDB muscle showed no motor unit action potential, while the left EDB muscle showed a reduced recruitment pattern and large amplitude of motor unit action potential (Table 2). Additionally, there was no electrophysiological evidence of lumbar radiculopathy or motor neuron disease. Considering the profound amplitude drop compared to the mild onset latency delay, distal predominant bilateral peroneal neuropathies with axonal loss were suggested. The results of routine serologic tests were within the reference values. Direct DNA sequencing of the PMP22 gene was performed for differential diagnosis of hereditary neuropathy; however, no pathologic mutation was detected. Whole genome sequencing (WGS) was performed to obtain more detailed genomic information and determine the underlying cause of bilateral peroneal neuropathies. Through WGS, a pathogenic TMEM67 gene mutation, which is responsible for JS type 6, was detected, and the patient was accordingly diagnosed with JS (Fig. 2).

**Table 1:** Nerve conduction study results.

Nerve	Stimulation	Right			Left		
		Latency (ms)	Amplitude (mV)	CV (m/s)	Latency (ms)	Amplitude (mV)	CV (m/s)
**Motor**							
Peroneal (EDB recording)	Ankle	4.5	1.0	–	5.8	1.3	–
	Fibular head	10.2	0.6	52.8	10.7	1.2	58.2
Peroneal (TA recording)	Fibular head	2.4	3.8	–	2.8	3.2	–
	Popliteal fossa	3.1	3.7	76.8	4.4	2.9	55.7
Tibial	Ankle	3.1	38.3		3.3	47.0	
	Popliteal fossa	10.3	22.2	49.1	10.5	32.9	53.6

CV = conduction velocity, EDB = extensor digitorum brevis, TA = tibialis anterior.

**Table 2:** Needle electromyography study results.

Side	Muscle	IA	ASA	MUAP	Recruit pattern
Right	Paraspinalis (L5-S1)	Normal	None	Normal	Full
	Extensor digitorum brevis	Normal	++	No activity	No activity
	Abductor hallucis	Normal	None	Normal	Full
	Tibialis anterior	Normal	None	Normal	Full
	Gastocnemius (medial)	Normal	None	Normal	Full
	Peroneus longus	Normal	None	Normal	Full
	Vastus lateralis	Normal	None	Normal	Full
	Biceps femoris (short)	Normal	None	Normal	Full
Left	Paraspinalis (L5-S1)	Normal	None	Normal	Full
	Extensor digitorum brevis	Normal	++	Large (9 mV)	Decreased
	Abductor hallucis	Normal	None	Normal	Full
	Tibialis anterior	Normal	None	Normal	Full
	Gastocnemius (medial)	Normal	None	Normal	Full
	Peroneus longus	Normal	None	Normal	Full
	Vastus lateralis	Normal	None	Normal	Full
	Biceps femoris (short)	Normal	None	Normal	Full

ASA = abnormal spontaneous activity, IA = insertional activity, MUAP = motor unit action potential.

## 3. Discussion

Peroneal nerve compromise has various causes, including trauma, external compression, intraneural ganglion, and nerve tumor.^[9,10]^ However, in this case, the patient presented with no proper conditions that could cause bilateral peroneal neuropathy, except for JS itself. Although the patient refused to undergo leg or lumbar MRI to identify structural problems, we considered that these were unlikely to be the cause of the peroneal neuropathy.

JS is caused by mutations in genes that encode proteins that make up the basal body and mother centriole of the primary cilium, or that regulate the development and function of the primary cilium.^[11]^ A defect in the ciliary gene leads to malfunction of various organs, including the kidney, retina, and brain. Additionally, a recent in vivo study revealed that pathological changes due to ciliopathy not only occur in the CNS but also in the PNS.^[6]^ In a study using chicken embryos, the primary cilium played an important role in neural crest cells, sensory neurons, and boundary cap cells during early PNS formation. Moreover, silencing of the JS-related protein C5orf42 was found to create defects in axonal development at the dorsal root ganglion of sciatic nerves, providing further evidence that the primary cilium participates in PNS development.^[6]^ Likewise, an association between ciliopathy and PNS dysfunction has been suggested, but a relationship between JS and PNS dysfunction has not been reported in clinical settings. To date, only CNS symptoms have been reported in JS. Therefore, the presented case is noteworthy because it represents the first report of PNS symptoms in JS. In JS, gait disturbance usually occurs due to atrophy of the cerebellum; therefore, it is thought that most previous studies have not conducted additional PNS evaluation. Examination of peripheral nerve function is limited due to the nature of the disease, which has a poor prognosis from a young age. We recommend conducting further assessments of PNS function in adult patients with JS, with the aim to shed light on the relationship between JS and PNS.

**Figure 3.**
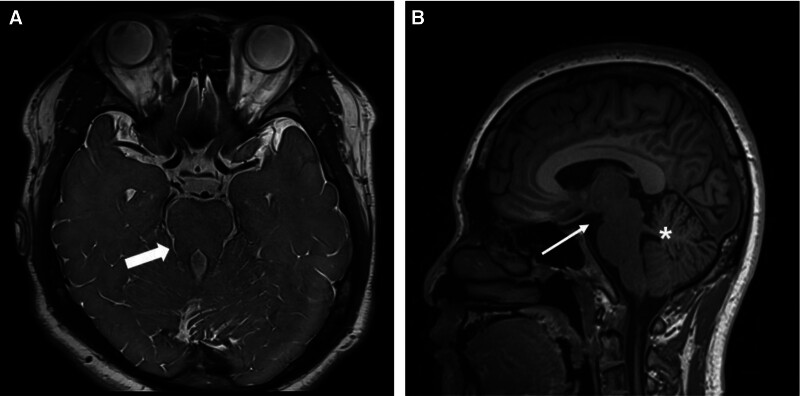
Brain magnetic resonance images. (A) An axial T2 image indicating mildly elongated superior cerebellar peduncles (thick arrow) and (B) a sagittal T2 image visualizing normal interpeduncular fossa depth (thin arrow) with no prominent vermian hypoplasia (asterisk).

JS presents with various clinical manifestations depending on its subtype. Despite this heterogeneity, the “molar tooth sign” represents a characteristic commonality. This molar tooth sign was established by combining radiological findings from brain MRI, such as lengthening and thickening of the superior cerebellar peduncle, deepening of the interpeduncular fossa, and hypoplasia of the cerebellar vermis. Although the molar tooth sign is considered an absolute diagnostic marker for JS, a few genetically confirmed cases of JS with normal or near-normal brain morphology have been reported.^[7]^ This indicates that the typical molar tooth sign does not appear in certain patients with JS with mild clinical symptoms. In addition, only mild elongation of the superior cerebellar peduncles was observed in the brain MRI of the presented case (Fig. 3). Because there was no radiologic hallmark of JS, the diagnosis was made genetically rather than radiologically. Therefore, contrary to previous consensus, JS cannot be completely dismissed even if the molar tooth sign is not detected on brain MRI. If JS is clinically suspected, genetic testing, such as WGS, may be helpful when the molar tooth sign is not observed on brain imaging. JS is a rare genetic disease with a prevalence of approximately 1:80,000 to 1:100,000,^[3]^ although this may be an underestimation due to some patients having mild symptoms.^[11]^ A genetic study confirming the etiology in patients with mild and ambiguous clinical features would likely unveil additional JS cases.

In conclusion, this report describes a case of JS with PNS abnormalities. To the best of our knowledge, this is the first case indicating an association between JS and peripheral nervous dysfunction in humans. Additional research is needed on peripheral nerve involvement in JS. Detailed genetic testing such as WGS could provide a diagnosis of atypical JS with normal or near-normal brain MRI findings.

## Acknowledgments

The authors thank the patient for agreeing to participate in this research.

## Author contributions

**Conceptualization:** Hyeong-Min Kim.

**Data curation:** Hyeong-Min Kim.

**Formal analysis:** Min-Keun Song, Hyeng-Kyu Park.

**Funding acquisition:** Min-Keun Song, Hyeng-Kyu Park.

**Investigation:** Hyeong-Min Kim.

**Methodology:** Hyeong-Min Kim.

**Project administration:** Min-Keun Song, Hyeng-Kyu Park.

**Resources:** Min-Keun Song, Hyeng-Kyu Park.

**Supervision:** Min-Keun Song, Hyeng-Kyu Park.

**Validation:** Min-Keun Song, Hyeng-Kyu Park.

**Visualization:** Min-Keun Song, Hyeng-Kyu Park.

**Writing – original draft:** Hyeong-Min Kim.

**Writing – review & editing:** Hyun-Seok Jo, Jae-Young Han, In-Sung Choi, Min-Keun Song, Hyeng-Kyu Park.
